# The *Plasmodium* transmission-blocking symbiont, *Microsporidia MB*, is vertically transmitted through *Anopheles arabiensis* germline stem cells

**DOI:** 10.1371/journal.ppat.1012340

**Published:** 2024-11-11

**Authors:** Thomas Ogao Onchuru, Edward Edmond Makhulu, Purity Cassandra Ronnie, Stancy Mandere, Fidel Gabriel Otieno, Joseph Gichuhi, Jeremy Keith Herren

**Affiliations:** 1 International Centre of Insect Physiology and Ecology (ICIPE), Kasarani, Nairobi, Kenya; 2 Department of Physical and Biological Sciences, Bomet University College, Bomet, Kenya; 3 Wits Research Institute for Malaria, University of the Witwatersrand, Witwatersrand, South Africa; University of Sao Paulo, BRAZIL

## Abstract

*Microsporidia MB* is a promising candidate for developing a symbiont-based strategy for malaria control because it disrupts the capacity of *An*. *arabiensis* to transmit the *Plasmodium* parasite. The symbiont is predominantly localized in the reproductive organs and is transmitted vertically from mother to offspring and horizontally (sexually) during mating. Due to the contribution of both transmission routes, *Microsporidia MB* has the potential to spread through target vector populations and become established at high prevalence. Stable and efficient vertical transmission of *Microsporidia MB* is important for its sustainable use for malaria control, however, the vertical transmission efficiency of *Microsporidia MB* can vary. In this study, we investigate the mechanistic basis of *Microsporidia MB* vertical transmission in *An*. *arabiensis*. We show that vertical transmission occurs through the acquisition of *Microsporidia MB* by *Anopheles* cystocyte progenitors following the division of germline stem cells. We also show that *Microsporidia MB* replicates to increase infection intensity in the oocyte of developing eggs when mosquitoes take a blood meal suggesting that symbiont proliferation in the ovary is coordinated with egg development. The rate of *Microsporidia MB* transmission to developing eggs is on average higher than the recorded (mother to adult offspring) vertical transmission rate. This likely indicates that a significant proportion of *An*. *arabiensis* offspring lose their *Microsporidia MB* symbionts during development. The stability of germline stem cell infections, coordination of symbiont proliferation, and very high rate of transmission from germline stem cells to developing eggs indicate that *Microsporidia MB* has a highly specialized vertical transmission strategy in *An*. *arabiensis*, which may explain host specificity.

## Author summary

Mosquito vectors of diseases are associated with a broad range of microbes. Some of the microbes significantly affect vector biology including pathogen transmission efficiency. *Anopheles* mosquitoes, which transmit the malaria parasite, *Plasmodium falciparum*, harbor a native microbe known as *Microsporidia MB*. This microbe interferes with the formation of transmissible stages of the parasite (sporozoites) that are transferred to humans by female mosquitoes when taking a blood meal. This phenotype can be exploited to develop a novel strategy for controlling malaria similar to the control of dengue fever using *Aedes* mosquitoes carrying *Wolbachia* bacteria. Mother to offspring transmission of protective microbes is important in sustainable application of microbe-based technologies to control vector-borne diseases because it ensures maintenance of the microbe in target vector populations across many generations. Here, we investigated stability of *Microsporidia MB* infections and efficiency of mother to offspring transmission during early stages of egg formation and development. We found that this microbe has a specialized transmission mechanism that involves infecting the germline cells that are important in egg production. We also demonstrated a very high transmission rate (97%) of the *Microsporidia MB* from infected germline cells into daughter cells during cell division. As the germline daughter cells developed into eggs, *Microsporidia MB* established itself in the egg yolk through active replication which only occured after the female mosquitoes had a blood meal. Our study gives insights into an efficient mother to offspring transmission route of *Microsporidia MB* that can be utilized sustainably in microbe-based intervention to control malaria.

## Introduction

Vector-borne diseases, and in particular malaria, are of global importance because of the associated economic burden and high mortality rates, especially in developing countries [1–3]. Malaria is transmitted by *Anopheles* mosquitoes and is responsible for over half a million deaths annually [3]. The primary method for controlling this disease is vector management which includes the use of long-lasting insecticidal nets (LLINs), indoor residual spraying (IRS), and the management of mosquito breeding sites [4]. The application of these methods has significantly reduced transmission intensity and subsequently the malaria burden. However, the progress in malaria control has stalled suggesting that the current malaria control strategies have limitations in driving malaria cases to significantly low levels for successful elimination [3,5]. Therefore, the development of novel approaches is urgently needed to sustain the fight against malaria.

There are now several cases where it has been demonstrated that microbes associated with mosquito vectors can significantly alter their ability to efficiently transmit pathogens [6]. For instance, the vectorial capacity of the *Aedes aegypti* mosquito, which is responsible for the transmission of medically important viruses such as dengue, chikungunya, and zika, is significantly impaired in the presence of an intracellular endosymbiont, *Wolbachia* [6]. Based on this phenotype and the ability of *Wolbachia* to spread through insect populations, *Wolbachia-*based strategies have been developed and implemented to control dengue fever transmission successfully [7–9]. Similarly, it has been demonstrated that the association of malaria-transmitting mosquitoes and specific microbial symbionts reduces the ability of these vectors to transmit malaria-causing *Plasmodium* parasites [10–14]. The efficiency of malaria transmission-blocking and the stability of association with the vector are important parameters to consider when selecting a potential symbiont for malaria control. Notably, endosymbiotic microbes that have high-fidelity vertical transmission have an inherent ability to maintain their prevalence across mosquito generations and are therefore likely to be sustainable and cost-effective as malaria transmission blockers. Recently, *Microsporidia MB*, was discovered as an endosymbiont of *Anopheles* mosquitoes and shown to impair the transmission of the *Plasmodium falciparum* parasite by *An*. *arabiensis* mosquito [14]. The exact mechanisms involved in transmission blocking have not yet been characterized. The fastidious nature of endosymbionts presents challenges to establishing the precise molecular mechanisms behind pathogen protection. In other insect-symbionts systems where the mechanistic basis of protection has been elucidated, studies have shown that protection can involve the production of antagonistic compounds, competition for nutritional resources and space, and host immune priming [15]. Unlike many previously reported microsporidians which are pathogens of mosquitoes [16], *Microsporidia MB* does not have any apparent negative fitness effects on its host [14] and is located in a different group, clade IV, which is distinct from other microsporidia clades containing most of the known parasitic microsporidians of mosquitoes [17]. *Microsporidia MB* is vertically transmitted (mother to offspring) and horizontally transmitted (sexually) and therefore able to spread efficiently through mosquito populations [14,18]. Recently, we have shown that *Microsporidia MB* is predominantly localized in the reproductive organs which is relevant for vertical transmission from mother to offspring and horizontal transmission during mating [18–20]. However, the precise mechanisms involved in the vertical transmission of *Microsporidia MB* have not been elucidated. Understanding the strategies employed by this symbiont to vertically infect *Anopheles* mosquitoes will be important to determine the factors limiting infection rates and specificity to different malaria vector species and populations. Understanding these factors could facilitate strategies to sustainably increase *Microsporidia MB* infections in natural populations to significantly reduce malaria infections.

In this study, we investigated how the microsporidian symbiont is transferred from mother to offspring by studying its localization within the cells of the *Anopheles* mosquito ovary. We report that *Microsporidia MB* is present in the germarium and infects the germline stem cells from where it enters the cystocytes as a result of stem cell division. As the cystocytes develop into follicles, *Microsporidia MB* infection intensities increase first in the nurse cells and then in oocytes through active symbiont cell division. The average rate of transmission from stem cells to follicles (>96%) is higher than the average rate of observed adult to adult vertical transmission, suggesting that some *Anopheles* mosquitoes lose their symbiont infections over development.

## Results

### *Microsporidia MB* is vertically transmitted in the *An*. *arabiensis* germarium

**Fig 1:**
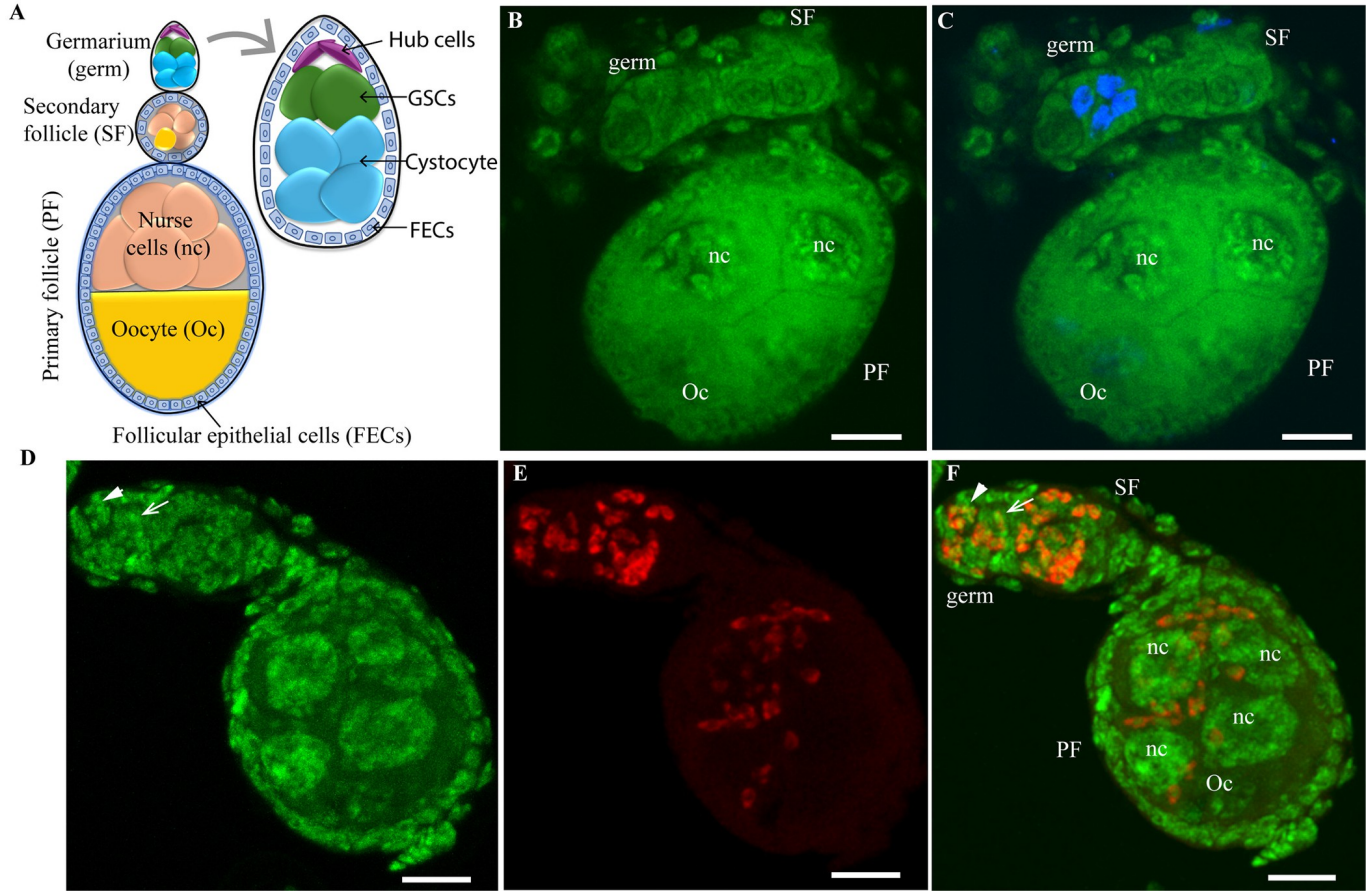
*Microsporidia MB* localization in the polytrophic *An*. *arabiensis* ovariole. (A) Schematic representation of the ovariole structure showing the germarium, the secondary follicle, and the primary follicle (with the oocyte and nurse cells in one chamber surrounded by the epithelial follicular cells). The germline stem cells (GSCs) and cystocytes are located in the germarium. (B & C) Images of a *Microsporidia MB* negative control ovariole obtained from a two-day-old *An*. *arabiensis* mosquito showing the localization of stem cell daughter cells, cystocytes (in blue), following cell divisions within the germarium (the blue signal represents newly synthesized DNA of cystocytes as detected by Click-iT Edu staining). (D-F) Images of a *Microsporidia MB-*infected *An*. *arabiensis* ovariole showing the distribution of the symbiont within the ovariole. *Microsporidia MB* is present in the germarium (germ) inside the GSCs (white arrowheads pointing to the GSC nucleus) and cystocytes (white arrow pointing to the cystocyte nucleus), the secondary follicle (SF), and the primary follicle (PF) i.e., the nurse cells (nc) and the oocyte (Oc). (Green represents Sytox Green DNA staining, image D, red represents *Microsporidia MB-*specific FISH probe staining, Image E, and a merged image of the green and red channel, Image F). Scale = 10μm. (Images are representative pictures of >100 independent observations).

*Microsporidia MB* was localized in *An*. *arabiensis* female ovaries using fluorescence in situ hybridization (FISH) microscopy with a *Microspordia MB*-specific probe [14]. *An*. *arabiensis* ovaries comprise tens to hundreds of polytrophic ovarioles with each ovariole composed of a germarium, the secondary, and the primary follicles. The germarium is located at the distal tip of the ovariole and contains 2–4 germline stem cells (GSCs). As each GSC divides, it produces an identical GSC and differentiating germline cell called a cystocyte. As a cystocyte progresses through the germarium, it undergoes mitotic divisions in combination with incomplete cytokinesis resulting in a follicle that has seven nurse cells connected through cytoplasmic bridges and one oocyte (Fig 1A and 1B). Through the detection of newly synthesized DNA, we found evidence of newly formed cystocytes following stem cell divisions in the germarium of newly emerged two-day-old mosquitoes (Fig 1C). We further demonstrated the localization of *Microsporidia MB* in the germarium (Figs 1D–1F and S1), inside the germline stem cells from where it is transferred to cystocytes during cell division. We also found *Microsporidia MB* infections in the secondary and primary follicles (Figs 1D–1F and S1). In the primary follicles, the symbiont was present in the developing oocytes and the nurse cells in high abundance but not in the follicular epithelial cells (Figs 1D–1F and S1).

### *Microsporidia MB* intensities increase in the oocyte during egg development and maturation

**Fig 2:**
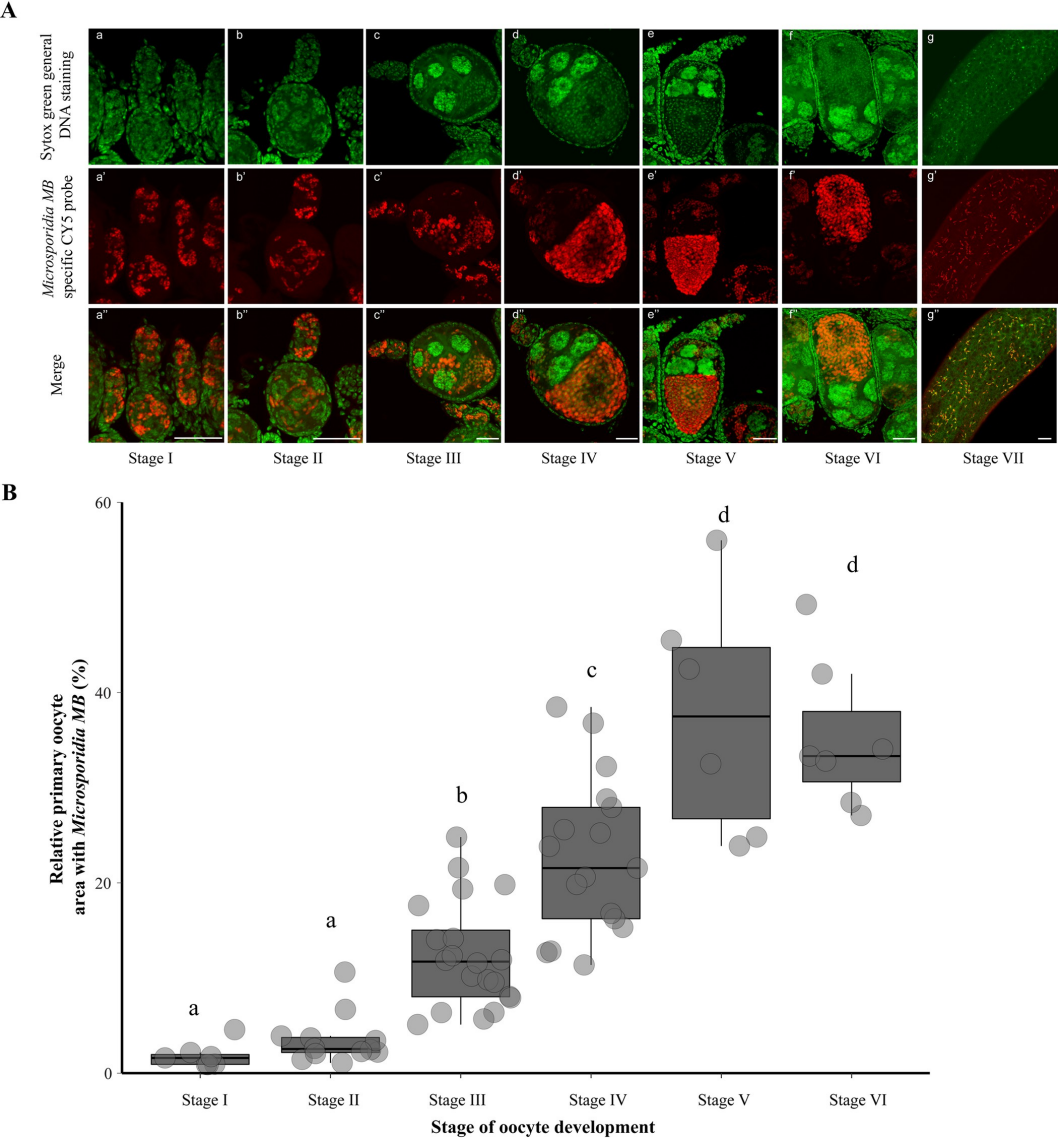
Localization of *Microsporidia MB* within the germarium and follicles across the different developmental stages of *An*. *arabiensis* ovariole. (A) Images showing *Microsporidia MB* localization in the germarium, secondary, and primary follicles in all ovariole developmental stages. *Microsporidia MB* is spread throughout the primary follicle during the previtellogenic phase of development (stages I-III). As development progresses to the vitellogenic phase (stages III-VI), microsporidia symbiont cells accumulate in the anterior end of the primary follicle oocyte in high densities. The upper panels (a-g) represent the Sytox Green DNA channel signal, the second row of panels (a’-g’) represents *Microsporidia MB-specific* CY-5 channel signal and the panels in the third row (a”-g”) represent merged images of both signals. Scale = 25μm. (B) The relative area of the primary follicle oocyte occupied by *Microsporidia MB* symbiont as measured using the Sytox Green DNA channel signal. *Microsporidia MB* occupies a small area of the primary follicle oocyte in the previtellogenic stages of development and increases significantly during the vitellogenic stages of development (χ^2^ [5] = 57.13, p < 0.01).

Differentiation of the primary follicle into a mature egg within the ovariole in Anopheline mosquitoes is characterized by seven stages; stages I-III (previtellogenesis) and stages IV-VII (vitellogenesis). The vitellogenic stages IV-VII only occur after the female mosquito takes a blood meal. To determine *Microsporidia MB* growth dynamics in the ovariole during development, we performed FISH microscopy for ovarioles at different stages of development. We analyzed *Microsporidia MB* localization patterns in the germarium, the secondary follicle, and the primary follicle and found them to be infected across all ovariole developmental stages (Figs 2A, a-g and S2A). In the early previtellogenic stages (stages I and II), the symbiont was evenly distributed in the nurse cells and oocyte of the primary follicle (Fig 2A, a and b). In the late previtellogenic stage and vitellogenic (stages III-VI), however, *Microsporidia MB* accumulated in the developing oocyte but decreased in the nurse cells (Fig 2A, c-f). Once the egg developed to maturity and established a typical cigar shape following the degeneration of the nurse cells, an even distribution of *Microsporidia MB* cells throughout the egg was observed (Fig 2A, g).

We also quantified relative *Microsporidia MB* infection intensities in the oocytes of ovarioles at different developmental stages by measuring the area covered by *Microsporidia MB* signal (based on SytoxGreen general DNA staining and *Microsporidia MB-*specific probe staining) in relation to the entire area covered by the primary follicle oocyte. Both measurements showed low *Microsporidia MB* infection intensities in previtellogenic stage oocytes (Figs 2B and S2B). However, the infection intensities exponentially increased during vitellogenic stages of ovariole development. (χ^2^ [5] = 57.13, p < 0.01). Previously, we observed a similar increase in *Microsporidia MB* infection intensities based on qPCR quantifications in *An*. *arabiensis* vitellogenic ovaries following blood feeding [20]. This increase of *Microsporidia MB* intensities inside the primary follicle oocyte suggests active proliferation of the symbiont or its deposition from the nurse cells.

### *Microsporidia MB* lifecycle and proliferation in the oocyte

**Fig 3:**
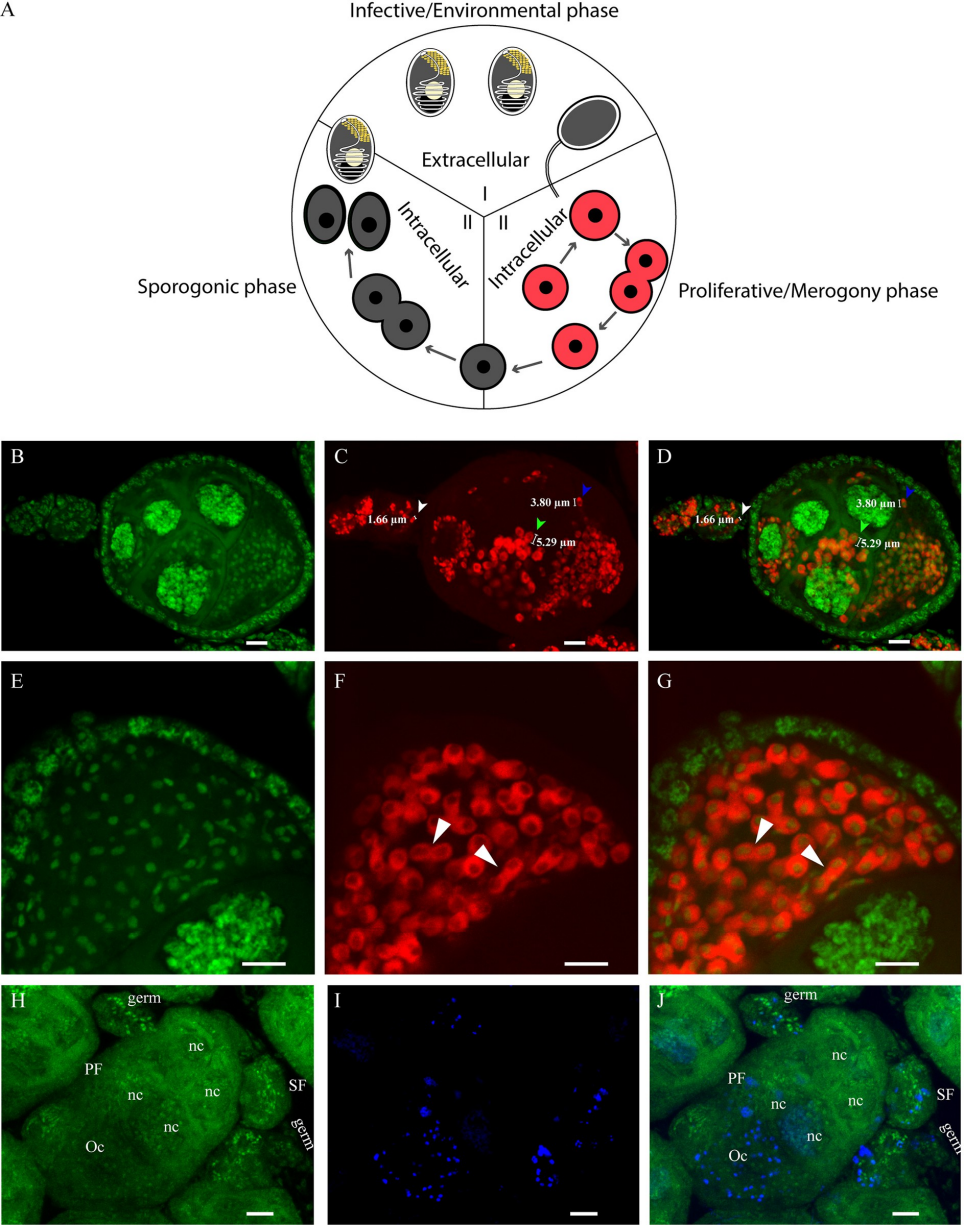
*Microsporidia MB* development in the ovariole. (A) A typical microsporidia lifecycle showing the three microsporidian developmental stages i.e., the infective stage, proliferative, and sporogonic stages (figure adapted from [21]). (B-H) Confocal microscopy images showing the developmental stages of *Microsporidia MB* observed in infected *An*. *arabiensis* ovarioles. (B-D) Images of an ovariole with different sizes of *Microsporidia MB* cells ranging from 2μm in the germarium and secondary follicle (white arrowhead) to 5μm in the nurse cells and oocyte of the primary follicle, green and blue arrowheads, respectively. (E-G) Images showing *Microsporidia MB* cells in the merogony stage of proliferation in the oocyte (white arrowheads) and the presence of newly synthesized DNA in actively proliferating cells (H-J). The first column (panels B, E, and H) in green represents the Sytox Green DNA staining signal, the second column (panels C and F) in red represents the *Microsporidia MB-specific* CY-5 staining signal while (panel I) in blue represents Click-iT Edu staining signal and the third column (panels D, G, J) represent merged images of two channels. Scale = 10μm.

We observed an increase of *Microsporidia MB* intensities in oocytes of the primary follicles across the vitellogenic developmental stages (Fig 2). To establish how *Microsporidia MB* intensities increase in the developing primary follicles, we investigated the physiological states of *Microsporidia MB* cells in the oocyte and compared them to typical microsporidian developmental stages which are mainly divided into three stages i.e., infective, proliferative, and sporogonic stages (Fig 3A) [21]. The infective, also known as the environmental phase, is the only extracellular part of the cycle where microsporidia exist only as inactive spores until they inject their sporoplasm into a host cell to establish an infection. The proliferative stage involves the intracellular division of microsporidia cells through the process of merogony. The cells then enter the sporogonic developmental stage to form spores that are eventually released into the environment. Using the *Microsporidia MB-*specific FISH probe, we identified different sizes of *Microsporidia MB* cells inside the primary follicles ranging from smaller cells with a size of about 2μm to larger cells with a size of about 5μm (Fig 3B–3D and S3 Fig) suggesting that the symbiont exists in multiple forms in the follicle. We also observed proliferative *Microsporidia MB* cells in the process of fission (merogony) in the oocyte (Fig 3E–3G), which indicated that the symbiont was actively proliferating within the developing oocyte. The pattern of *Microsporidia MB* development in the *Anopheles* oocyte is consistent with merogony since intracellular proliferation was observed and spores were not detected. To confirm *Microsporidia MB* proliferation in the ovariole, we stained for actively dividing *Microsporidia MB* cells in infected ovarioles using Click-iT EdU cell proliferation assay that detects a nucleus with newly synthesized DNA following cell division. *Microsporidia MB* nuclei containing newly synthesized DNA can be differentiated from the mosquito’s newly synthesized nuclei (as observed in Fig 1C) because of its small size which is consistent with the Sytox Green DNA staining. Using the Click-iT EdU cell proliferation assay we were able to detect new *Microsporidia MB* cells mostly in the oocyte of the primary follicle but also the secondary follicle and the germarium (Fig 3H–3J) of infected ovarioles.

### Vertical transmission rate of *Microsporidia MB* into primary follicles

**Fig 4:**
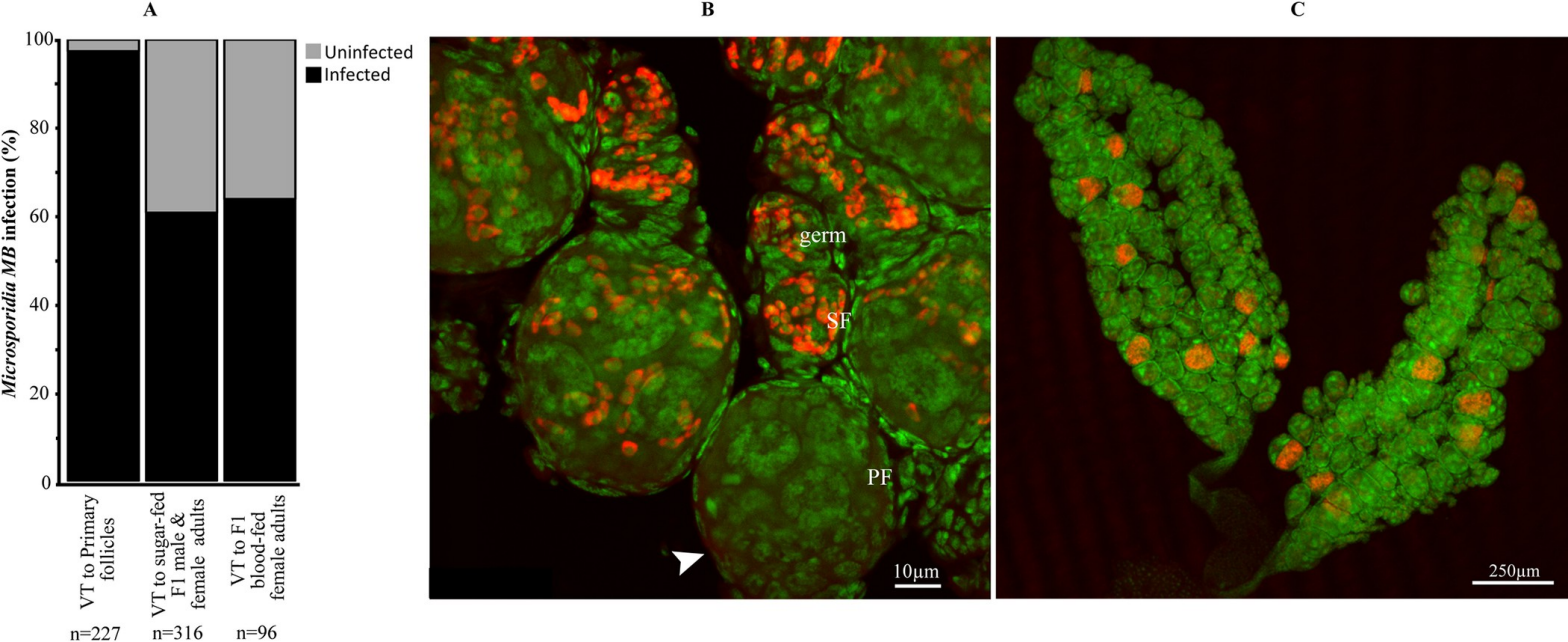
*Microsporidia MB* infection rates in primary follicles and vertical transmission (VT) rates. *Microsporidia MB* distribution in infected female ovaries was investigated and compared to vertical transmission rates. (A-B) A majority (97%) of the primary follicles within infected ovarioles had *Microsporidia MB* while the symbiont was completely absent in 3% of the primary follicles (B, white arrowhead). However, the germarium (germ) and secondary follicles (SF) of the 3% primary follicles that lacked *Microsporidia MB* were consistently infected with *Microsporidia MB*. *Microsporidia MB* was detected in 61% of sugar-fed (male & female) and 63% of blood-fed (female) adult offspring of *Microsporidia MB-*infected mothers. (C) *Microsporidia MB* was found to be present in both ovaries of infected female gonads investigated. Green represents Sytox Green general DNA staining while red represents *Microsporidia MB-specific* CY-5 probe staining.

We quantified *Microsporidia MB* infection rates of primary follicles in the ovaries to establish the vertical transmission rate of *Microsporidia MB* into developing eggs in infected ovaries. We found that a small number of primary follicles (3%) were not infected with *Microsporidia MB* while a majority (97%) of the primary follicles in infected ovaries had *Microsporidia MB* (Fig 4A). Interestingly, in ovarioles whose primary follicles were not infected with *Microsporidia MB* (3%), the symbiont was consistently present in the germarium and the secondary follicles (Fig 4B) suggesting that early stem cell divisions could occasionally not transfer *Microsporidia MB* to the follicles. To establish whether this phenomenon could explain the imperfect vertical transmission rate (45–100%) of *Microsporidia MB* [10], we compared the primary follicle infection rate and *Microsporidia MB* prevalence in sugar-fed adult F1 offspring (males and females) and blood-fed adult F1 offspring (females) obtained from *Microsporidia MB*-infected isofemale mothers. We found that whereas 97% of the primary follicles were infected with *Microsporidia MB*, only 61% of sugar-fed adult F1 offspring and 63% of blood-fed adult F1 females were infected with *Microsporidia MB* (Fig 4A), suggesting that *Microsporidia MB* prevalence is not influenced by nutritional status in adults and is likely lost during pre-imaginal stages of development. In all *Microsporidia MB-*infected *An*. *arabiensis* female gonads we investigated, we found the symbiont to be localized in both ovaries indicating even distribution of the symbiont in infected gonads (Fig 4C).

## Discussion

Successful deployment of a novel *Microsporidia MB*-based approach for controlling malaria will depend on the ability of the microsporidian symbiont to invade, spread, and be maintained in target *An*. *arabiensis* vector populations. While *Microsporidia MB* has high rates of maternal transmission in *An*. *arabiensis*, these rates are variable. The rate of maternal transmission is critical for determining the prevalence of *Microsporidia MB* in a population of *Anopheles* mosquitoes and the impact on *Plasmodium* [22]. In this study, we established the mechanistic basis of *Microsporidia MB* vertical transmission, which occurs through the acquisition of *Microsporidia MB* by *Anopheles* cystocyte progenitors from the germline stem cells following cell division. As the daughter stem cell cystocytes differentiate into follicles, *Microsporidia MB* establishes itself in the nurse cells and ultimately the oocyte and mature egg. Our findings suggest that *Microsporidia MB* becomes established in the *Anopheles* germline early during development and this seems to be highly efficient, as only 3% of primary follicles do not acquire a *Microsporidia MB* infections. We didn’t find any evidence of the germline becoming re-infected from somatic cell infections, but we do not rule out that this could be occurring. In particular, we have previously observed that *Microsporidia MB* infections acquired via sexual transmission can subsequently be vertically transmitted [18]. Vertical transmission of microsporidian symbionts through egg infections has been reported in different species of mosquitoes and in the amphipod *Gammarus duebeni* [23–27]. However, symbiont infections in these hosts were only observed in the oocyte and epithelial follicular cells but not in the germline stem cells suggesting that the mechanism of vertical transmission is through somatic cell infections and not germline stem cell infections as we found for *Microsporidia MB*. Somatic cell infections and subsequent transovarial transmission has been demonstrated for *Edhazardia aedis* in *Aedes aegypti* and *Amblyospora* sp. in *Aedes cantator*, *Aedes triseriatus* and *Culex salinarius* when horizontally (orally) acquired spores infect oenocytes and spread to the ovaries where they proliferate and form transovarial spores [16,23,25,28]. Similarly, vertical transmission of a microsporidian symbiont of *Gammarus duebeni* occurs when the symbiont invades and proliferates in follicular cells before spreading into the developing oocytes for transmission to offspring [24].

The mechanistic basis of *Microsporidia MB* transmission in *An*. *arabiensis* is strikingly similar to *Wolbachia* infections in other insects including *D*. *melanogaster* and *Aedes* mosquito species where the endosymbiont is in the germline stem cells and is vertically transferred to the progeny following stem cell divisions [12,29–31]. For *Wolbachia*, there is apparently an additional vertical transmission route, which involves infection of the follicle from the surrounding somatic cells. This additional infection route may be important for maintaining near-perfect rates of vertical transmission, which are required for an endosymbiont that is entirely dependent on vertical transmission [31,32]. In this study, *Microsporidia MB* was not observed in the somatically derived epithelial follicular cells that surround the egg chamber. *Wolbachia* infections of the germline stem cells of *Drosophila* are capable of influencing mitotic processes to promote more egg production [33,34]. Although *Microsporidia MB* does not apparently affect egg production in *An*. *arabiensis* [14], its high infection intensity in the germline stem cells and developing oocytes may have consequences for cellular processes and embryonic development. It is notable that the mechanistic basis of *Microsporidia MB* vertical transmission is very different from another insect endosymbiont, *Spiroplasma*. *Spiroplasma* is absent from the germline stem cells but gains access to the vitellogenic oocyte during vitellogenesis by co-opting the yolk transport system [35]. The existence of *Microsporidia MB* in the stem cells and oocyte of developing eggs in *An*. *arabiensis* provides strong evidence for a highly specialized vertical transmission strategy for *Microsporidia MB* in *An*. *arabiensis*. This high level of specialization may explain why *Microsporidia MB* has so far only been found in *Anopheles* mosquitoes [18].

Similar to other microsporidians, *Microsporidia MB* is a unicellular obligate intracellular organism that can only replicate and survive inside the host cells and only exist as spores outside the host cells [36]. Our findings demonstrate that cystocytes, nurse cells, and oocytes are among the suitable host cell types for this symbiont within the *An*. *Arabiensis* host. We identified different sizes of *Microsporidia MB* cells in the ovariole suggesting that *Microsporidia MB* is transitioning through different life cycle stages in this tissue. Further, we found evidence that *An*. *arabiensis* ovarioles provide an optimal environment for the proliferative phase of *Microsporidia MB* development. We observed that this microsporidian symbiont actively proliferates inside the primary follicle oocyte through the detection of *Microsporidia MB* cells undergoing merogony and the presence of newly synthesized *Microsporidia MB* DNA. This proliferation was observed after mosquitoes had a successful blood meal suggesting that *Microsporidia MB* proliferation is coordinated with host cellular processes. It is notable that in a typical microsporidian lifecycle, sporogeny follows merogony. In the case of *Microsporidia MB*, it is not clear if sporogeny plays a role in vertical transmission since spores enable survival in the environment and invasion of cells and we observe that vertical transmission occurs without *Microsporidia MB* leaving an intracellular environment. Sporulation of *Amblyospora* species of microsporidia in *Culex salinarius* and *Culiseta incidens* only occurs after the mosquitoes take a blood meal and it coincides with the maturation of the oocytes [23,37]. The development of the microsporidian symbiont of *Gammarus duebeni* is also highly coordinated with the host reproductive cycle [24]. The controlled proliferation of symbionts within insect hosts is important in mitigating any fitness costs to the host [24,38,39]. Unlike other pathogenic microsporidian symbionts that kill their hosts [36], *Microsporidia MB* has no apparent fitness costs to the host despite attaining high infection intensities in the oocyte of the developing egg [14]. For vertically transmitted symbionts, there’s a strong selection for avirulence to the host because the symbiont’s success is associated with the host’s reproductive success [40,41]. Therefore, the specialized vertical transmission of *Microsporidia MB* through infection of oocytes of developing eggs of *An*. *arabiensis* suggests that avirulence has been selected for during evolution leading to its low pathogenicity to the host.

The presence of the microsporidian symbiont in most (97%) of the developing eggs implies a potential for high vertical transmission rates. However, in our experiments, we observed an average of 61% and 63% vertical transmission rates from infected mothers to sugar-fed and blood-fed adult F1s, respectively, which was lower than the infection rate in eggs that were developing in the ovary. Previously, we reported marginally higher transmission rates of *Microsporidia MB* from infected mothers to their offspring [14]. The absence of the symbiont in some of the primary follicles only partially explains the imperfect vertical transmission rate. Additionally, other factors such as the ability to gain access to target host cells and tissues (possibly the germline stem cells), host immune responses, and environmental factors during embryogenesis and larval stages may be responsible for the loss of infections during mosquito development. As demonstrated in other insect-symbiont associations [42–44], the presence of *Microsporidia MB* may elicit *An*. *arabiensis’* immune response which then directly interferes with the establishment of the symbiont before it colonizes the primary sites of infection where it may be protected from the host’s immune responses. Also, environmental factors such as temperature and humidity that are known to influence the occurrence and transmission of different endosymbionts including microsporidian symbionts in other insects are likely to affect the establishment of *Microsporidia MB* [45–47].

Our study has shown a specialized vertical transmission route of *Microsporidia MB* in *An*. *arabiensis* that is relevant in developing a symbiont-based strategy to control malaria. These findings will serve as a basis for investigations on *Microsporidia MB* transmission phenotypes in *Microsporidia MB* found in diverse geographical regions and Anopheline vector species aimed at maximizing *Microsporidia MB* vertical transmission rates for the development of a viable strategy for symbiont-based malaria transmission blocking.

## Methods

### Mosquito collection and rearing

Gravid *An*. *gambiae* s.l mosquitoes were collected from the Ahero irrigation scheme in Kenya in 2022 and 2023 and transported to icipe’s Duduville campus in Nairobi Kenya. The mosquitoes were then maintained in the insectary at 27 ± 2.5°C, humidity 60–80%, 12-h day and 12-h night cycles and induced to oviposit through the provision of oviposition cups with a wet cotton wool lined with a filter paper. After ovipositing, DNA was extracted from the females and used for species identification and screening for *Microsporidia MB* infections using PCR [14]. Offspring from *Microsporidia MB-*infected and uninfected female *An*. *arabiensis* mosquitoes were then reared in the insectary as described above for experimental investigations. Blood feeding of mosquitoes to initiate the processes of vitellogenesis and oogenesis was conducted as previously described [20].

### Fluorescence *in situ* hybridization (FISH) staining protocol

FISH was conducted to localize *Microsporidia MB* in the ovarioles of newly emerged 2-day-old and 2–5 days post-blood-fed *An*. *arabiensis* mosquitoes. Ovaries of *Microsporidia MB*-infected and uninfected (controls) were dissected in 1x PBS and fixed overnight in 4% Paraformaldehyde (PFA) at 4°C then rehydrated in 50% ethanol for 30 minutes, followed by a washing step in 1x PBS for 30 minutes. FISH was conducted as previously described to localize *Microsporidia MB* within the ovarioles [14,20]. Briefly, hybridization was done by incubating the tissues at 50˚C overnight in 100μl of hybridization mix (hybridization buffer i.e., dH2O, 5M NaCl, 1M Tris/HCl [pH = 8], and 10% SDS), 0.5μM final concentration of the *Microsporidia MB* specific CY5 probe (5′-CY5-CCCTGTCCACTATACCTAATGAACAT-3′) [10], and 0.025μM Sytox Green general DNA staining. After staining, the samples were washed twice with 100μl of wash buffer prewarmed at 50˚C (dH2O, 5M NaCl, 1M Tris/HCl [pH = 8], 0.5M EDTA, and 10% SDS). The tissues were subsequently mounted on glass slides using fluoromount mounting medium for confocal microscopy.

### Click-iT EdU staining of actively dividing cells in *An*. *arabiensis* germarium

Ovaries of *An*. *arabiensis* females were dissected in 10μM EdU in 1x PBS solution and labeled in this solution for one hour before they were fixed in a 3.7% PFA solution for 30 minutes. Subsequently, they were washed twice with 500μL of 3% BSA in 1x PBS and permeabilized using 0.5% Triton X-100 in 1x PBS for 20 minutes at room temperature before washing twice with 500μL of 3% BSA in 1x PBS solution. The ovaries were then stained and protected from light for 2 hours in a freshly prepared 500μL Click-iT Plus reaction cocktail prepared as per the manufacturer’s specifications. Once the Click-iT staining process was complete, the tissues were washed in with 500μL BSA in 1x PBS solution and counterstained with Sytox Green fluorescent general DNA stain (5 μg/ml) before mounting on a slide using fluoromount mounting medium for imaging.

### Confocal microscopy and image processing

The FISH and Click-iT EdU stained tissues were visualized immediately after mounting using a Leica SP5 confocal microscope (Leica Microsystems, USA), and images were taken using LAS AF software (Leica Microsystems, USA). Each stain was detected and images were captured independently using unique laser wavelength settings that were standardized across all samples. The images were analyzed using the ImageJ 1.50i software package [31] to identify *Microsporidia MB* and the host. *Microsporidia MB* was detected and quantified using a *Microsporidia MB* specific CY-5 FISH probe that targeted *Microsporidia MB* 18s rDNA and rRNA (red signal) while *Microsporidia MB* and *An*. *arabiensis* DNA in the nuclei was identified using Sytox Green general DNA stain (green signal). Based on multiple examinations of general DNA staining of *Microsporidia MB* infected and uninfected tissues, we identified the host cell nucleus from *Microsporidia MB* nucleus based on their size, location, and shape. This method has been used to identify *Wolbachia* in infected insect tissues [48]. Additionally, in all *Microsporidia MB* infected samples, there was a Sytox Green general DNA channel signal within the *Microsporidia MB* specific CY-5 FISH probe channel signal in the cell cytoplasm. This was used to establish the size and location of the *Microsporidia MB* nucleus and distinguish it from *An*. *arabiensis* nucleus. Subsequently, the distinct and consistent small size of *Microsporidia MB* nucleus in the cytoplasm as determined by the Sytox Green channel signal and *Microsporidia MB* specific probe staining was then used to distinguish between actively dividing *Microsporidia MB* and *An*. *arabiensis* cells after Click-iT Edu staining that targets nuclei with newly synthesized DNA.

### Quantification of *Microsporidia MB* in *An*. *arabiensis* ovaries

ImageJ software was used to measure the area occupied by *Microsporidia MB* in the primary follicle oocyte by measuring the fluorescence intensity signal captured by the *Microsporidia MB-*specific probe channel. Measurements based on the Sytox Green general DNA channel were also performed to avoid overestimating infection intensities using the *Microsporidia MB-*specific probe because it targets both 18s rDNA and 18s rRNA and therefore likely to be influenced by 18s rRNA expression patterns depending on the stage of *Microsporidia MB* development. The images acquired using the *Microsporidia MB-*specific probe channel and Sytox Green DNA channel were independently processed in ImageJ and then converted into an 8-bit grayscale binary scale. The regions of interest (ROI) around each of these structures were defined using the polygon selection tool in ImageJ based on the position of the follicular epithelial cells so that only the pixels within the area of interest were selected and included in the final measurements. Subsequently, we measured the proportion of the area that was occupied by *Microsporidia MB* cells within each of the ROIs as previously described [49]. The area occupied by the host nucleus was also calculated and removed from the total area obtained when the analysis was performed using the Sytox Green DNA signal channel that detects both *Microsporidia MB* and *An*. *arabiensis* DNA. Images where the symbiont and host nuclei staining were not clearly distinguishable were not included in the analysis. The relative oocyte area covered by *Microsporidia MB* across the different stages of egg development was then compared using Kruskal-Wallis H test. Rstudio software (2023.12.1.402) was used for statistical analysis [50].

### *Microsporidia MB* infection rates in the ovaries

To determine *Microsporidia MB* infection rates in the ovaries, a complete set of eight ovaries was obtained from select offspring of wild-caught *Microsporidia MB*-infected mothers that were collected from different locations within Ahero. The ovaries were stained by FISH as described above and the presence or absence of *Microsporidia MB* in both ovaries was recorded. *Microsporidia MB* infection rates in the primary follicles were determined by randomly selecting and counting the number of infected and uninfected follicles in the eight pairs of ovaries.

### Vertical transmission rate of *Microsporidia MB*

Field-collected gravid females were induced to oviposit as described above and then screened for *Microsporidia MB* infections using PCR [14]. Eggs from *Microsporidia MB-*infected mothers were hatched in the insectary and maintained at a temperature of 27 degrees and the larvae were fed on tetramin fish food until they pupated. The isofemale line pupae were subsequently transferred into cages where they emerged. We then performed two experiments to determine the vertical transmission rates. In one experiment, emerged F1s from 32 isofemale lines were maintained on a 10% sugar diet for two days and then immediately screened for *Microsporidia MB*. In another experiment, emerged F1s from 5 isofemale lines were maintained on a 10% sugar diet for two days and then given a blood meal for two consecutive days as previously described [20] before they were screened for *Microsporidia MB* 3 days post blood feeding. DNA was extracted from these two sets of experiments and used to screen for *Microsporidia MB* with PCR and qPCR as previously described [14,20]. We used controls, a plasmid construct with low copy number concentrations (10–100 copies) of the target gene to determine the detection limits of our qPCR protocol to ensure that low *Microsporidia MB* infections were measured. The number of infected adult offspring per isofemale line was expressed as a percentage.

## Supporting information

S1 Fig Representative microscopic images of *Microsporidia MB* localized in *An*. *arabiensis* ovariole. (A) *Microsporidia MB* negative control ovariole of a two-day-old *An*. *arabiensis* mosquito showing the localization of stem cell daughter cells, cystocytes, (in blue as detected by Click-iT Edu staining) within the germarium. (B) Image of a *Microsporidia MB* infected ovarioles of *An*. *arabiensis* mosquito showing localization of *Microsporidia MB* in the germline stem cells (white arrowheads) and daughter cystocyte cells (white arrows) in the germarium (germ), secondary follicles (SF) and the nurse cells (nc) and oocyte (oc) of the primary follicle (PF). (Green represents Sytox Green DNA staining, red represents *Microsporidia MB-*specific FISH probe staining). Scale = 10μm.
(TIF)

S2 Fig Representative microscopic images of *Microsporidia MB* localization during egg development. (A-G) Images of microscopic observations of *Microsporidia MB* in ovariolesduring egg maturation. (B) The relative area of the primary follicle oocyte occupied by *Microsporidia MB* symbiont as measured using the *Microsporidia MB* specific CY5 probe signal. *Microsporidia MB* occupies a small area of the primary follicle oocyte in the previtellogenic stages of development and increases significantly during the vitellogenic stages of development as observed with the Sytox Green DNA signal (χ^2^ [5] = 75.26, p < 0.01). Scale = 10μm.
(TIF)

S3 Fig Representative images of microscopic observations showing the different sizes of *Microsporidia MB* cells existing in the ovarioles ranging from 2μm in the germarium, secondary follicle, and oocyte (blue arrowheads) to 5μm in the nurse cells and oocyte of the primary follicle (white arrows). Green represents Sytox Green DNA staining signal, Red represents the *Microsporidia MB*-specific CY-5 staining signal, and blue Click-iT Edu staining signal. Scale = 10μm.
(TIF)
