# Supplementary figures and images for: The Plasmodium transmission-blocking symbiont, Microsporidia MB, is vertically transmitted through Anopheles arabiensis germline stem cells
Source: PLoS Pathog. 2024 Nov 11;20(11):e1012340. doi: 10.1371/journal.ppat.1012340 (PMC11581390; doi:10.1371/journal.ppat.1012340)

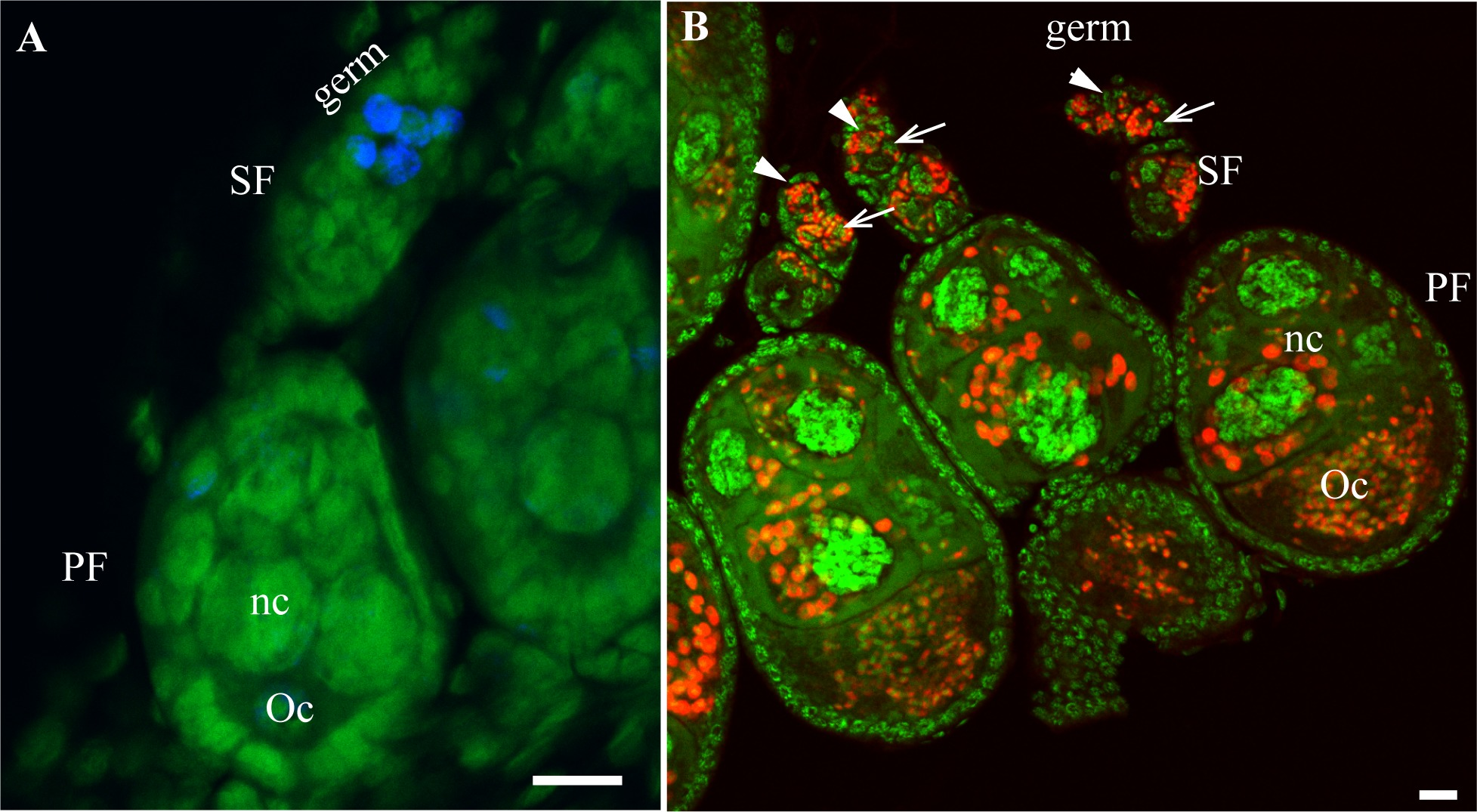

Supplement: S1 Fig — (A) Microsporidia MB negative control ovariole of a two-day-old An. arabiensis mosquito showing the localization of stem cell daughter cells, cystocytes, (in blue as detected by Click-iT Edu staining) within the germarium. (B) Image of a Microsporidia MB infected ovarioles of An. arabiensis mosquito showing localization of Microsporidia MB in the germline stem cells (white arrowheads) and daughter cystocyte cells (white arrows) in the germarium (germ), secondary follicles (SF) and the nurse cells (nc) and oocyte (oc) of the primary follicle (PF). (Green represents Sytox Green DNA staining, red represents Microsporidia MB-specific FISH probe staining). Scale = 10μm. (TIF) [file ppat.1012340.s001.tif]

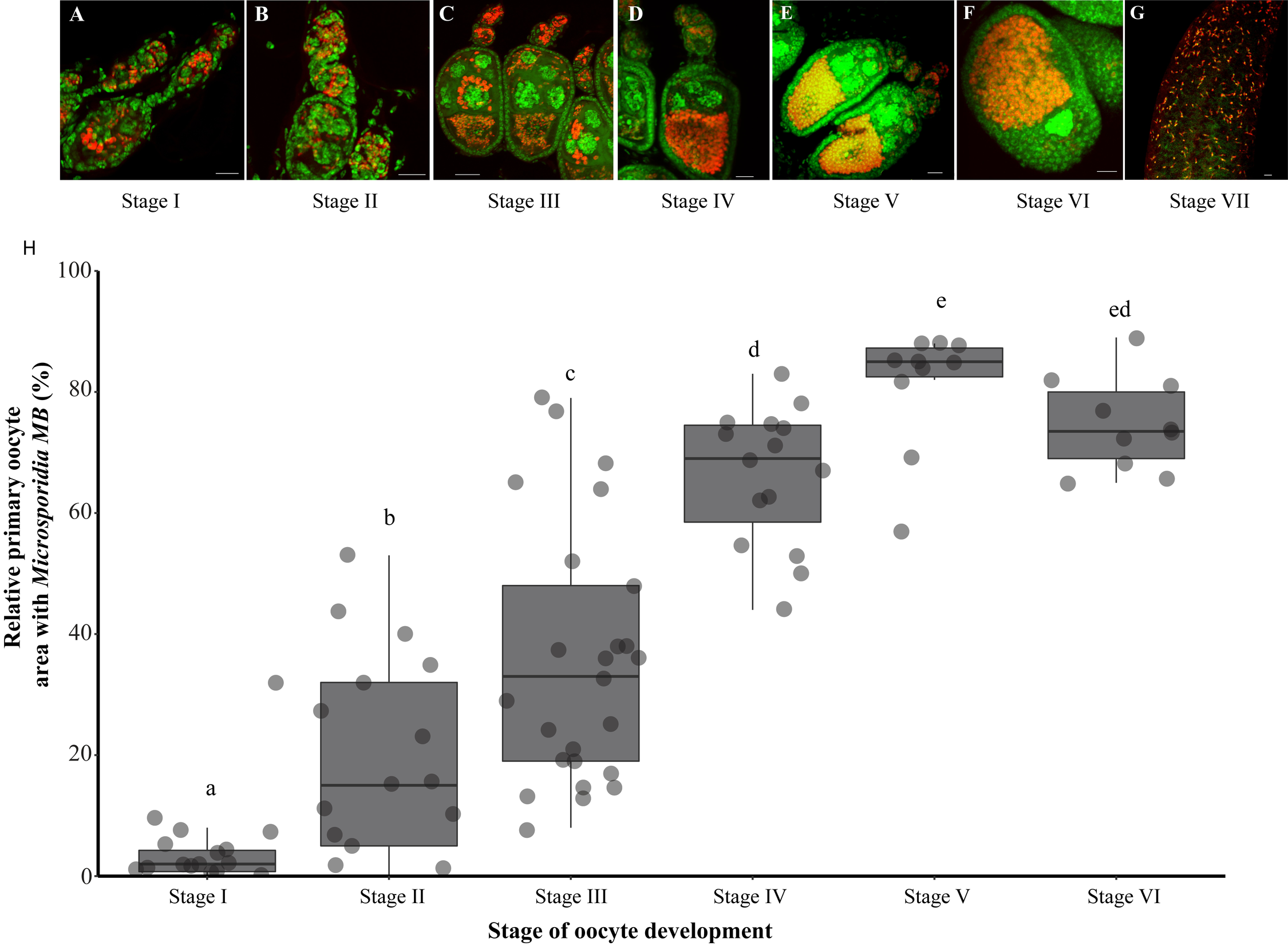

Supplement: S2 Fig — (A-G) Images of microscopic observations of Microsporidia MB in ovariolesduring egg maturation. (B) The relative area of the primary follicle oocyte occupied by Microsporidia MB symbiont as measured using the Microsporidia MB specific CY5 probe signal. Microsporidia MB occupies a small area of the primary follicle oocyte in the previtellogenic stages of development and increases significantly during the vitellogenic stages of development as observed with the Sytox Green DNA signal (χ2 [5] = 75.26, p < 0.01). Scale = 10μm. (TIF) [file ppat.1012340.s002.tif]

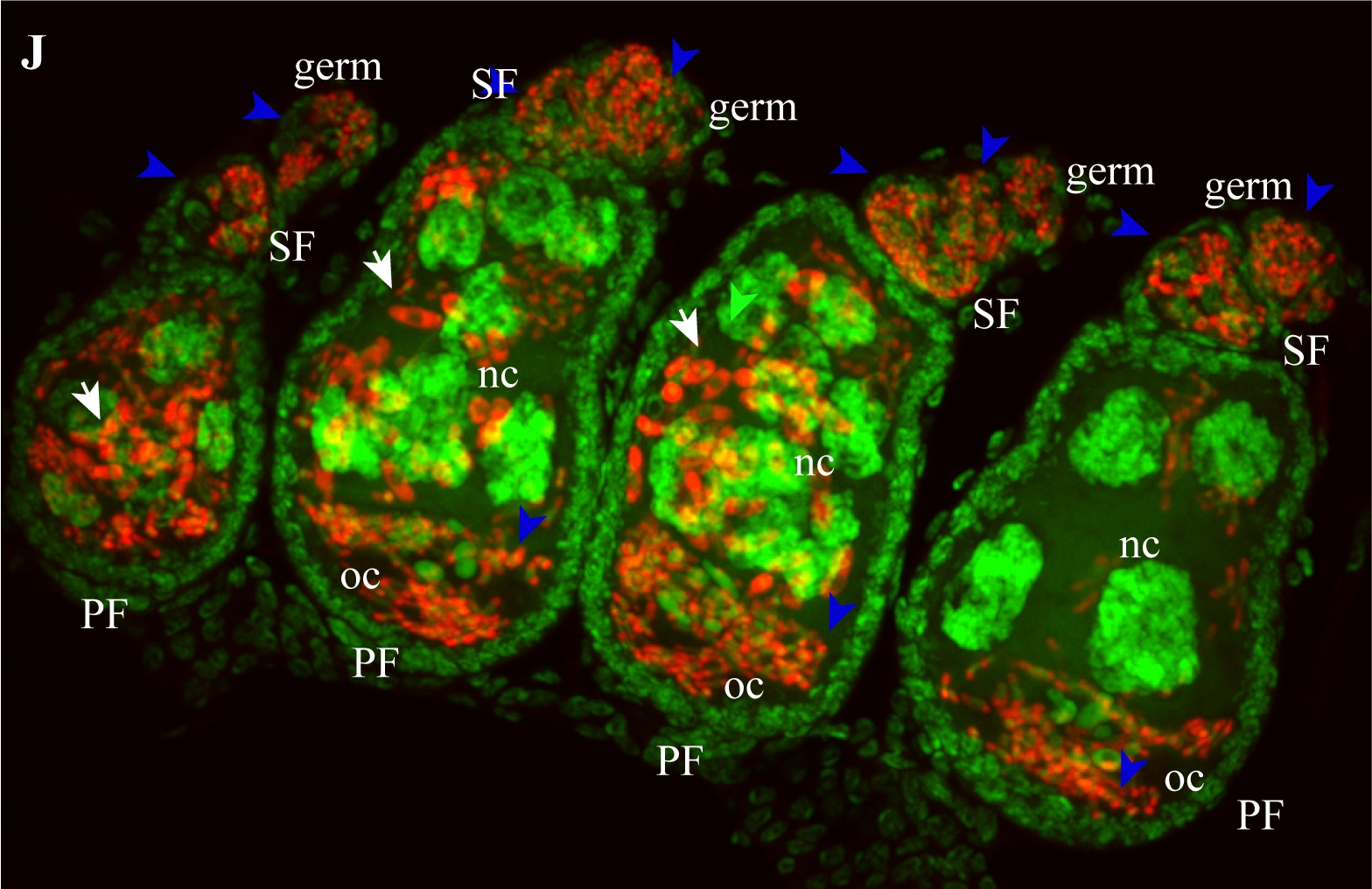

Supplement: S3 Fig — Green represents Sytox Green DNA staining signal, Red represents the Microsporidia MB-specific CY-5 staining signal, and blue Click-iT Edu staining signal. Scale = 10μm. (TIF) [file ppat.1012340.s003.tif]
